# EBV-positive diffuse large B-cell lymphoma in a human T-lymphotropic virus type 1 carrier

**DOI:** 10.1186/1750-9378-4-10

**Published:** 2009-07-06

**Authors:** Brady Beltran, Renzo Salas, Pilar Quiñones, Domingo Morales, Fernando Hurtado, Esther Cotrina, Luis Riva, Jorge Castillo

**Affiliations:** 1 Department of Oncology and Radiotherapy, Edgardo Rebagliati Martins Hospital, Lima, Peru; 2 Department of Pathology, Edgardo Rebaglati Martins, Lima, Peru; 3 Nursing Department, Edgardo Rebaglati Martins, Lima, Peru; 4 The Warren Alpert Medical School of Brown University, Division of Hematology and Oncology, The Miriam Hospital, Providence, RI, USA

## Abstract

The development of B-cell lymphomas has been seldom described in HTLV-1 carriers. We present the case of an elderly Peruvian HTLV-1 carrier who was diagnosed with EBV-positive diffuse large B-cell lymphoma. Despite an initial good response to therapy, patient died during treatment due to fatal *Pneumocystis jirovecci *pneumonia. EBV infection is characterized by B-cell lymphotropism and selective immunodeficiency. HTLV-1, on the other hand, induces T-cell dysfunction and B-cell proliferation. Finally, immunosenescence is characterized by T-cell dysregulation, decreased apoptosis and cytokine upregulation. In this elderly patient, the combination of EBV and HTLV-1 coinfection and immunosenescence may have played a role in the development of this aggressive diffuse large B-cell lymphoma. Furthermore, the immunodeficiency caused by the viral infections and chemotherapy may have played a role in developing life-threatening infectious complications.

## Findings

The Epstein Barr virus (EBV) was the first described oncovirus, which has been associated with the development of a variety of lymphoproliferative disorders, such as Burkitt [1], primary CNS [2], NK/T-cell [3], plasmablastic [4] and Hodgkin lymphoma [5]. EBV infection occurs early in childhood, and approximately 90 to 95% of adults worldwide are EBV-seropositive. EBV expression has also been reported in patients with diffuse large B-cell lymphoma (DLBCL) [6]. DLBCL is the most common variant of non-Hodgkin lymphoma in the United States (US) and accounts for approximately 25–30% of the cases [7]. In Peru, DLBCL accounts for up to 45% of all lymphomas and, akin to Asian countries, there is high incidence of T-cell lymphomas and low incidence of follicular lymphomas [8]. On the other hand, the human T-lymphotropic virus type 1 (HTLV-1) is a retrovirus and is the pathogenic agent of adult T-cell lymphoma/leukemia (ATLL) and other diseases [9]. HTLV-1 is endemic in Japan, the Melanesian Islands, the Caribbean, South America, the Middle East and parts of Africa. The prevalence of HTLV-1 in Europe and the US is lower than 1%. In Peru, it is estimated that up to 3% of the healthy adult population carry HTLV-1 [10]. The interaction of these two oncoviruses, EBV and HTLV-1, has seldom been reported in the medical literature.

The case is an 85-year-old Peruvian man with a past medical history of hypertension, who presented with a seven-week history of bilateral cervical node enlargement. The patient denied weight loss, drenching night sweats or fever. Physical examination showed an elderly individual with good performance status (ECOG 1) and non-tender bilateral cervical lymphadenopathy. No hepatosplenomegaly was found. CT scans of the neck, chest, abdomen and pelvis did not reveal other sites of disease. Complete blood count revealed 6,900 leucocytes per mm^3^, with 52% neutrophils and 28% lymphocytes; the white blood cell morphology was unremarkable. Hemoglobin was 13.6 g/dl and platelets 245,000 per mm^3^. Serum lactate dehydrogenase (LDH) levels were within normal limits. Renal and hepatic function tests and immunoglobulin A, G, M and E quantification were within normal ranges. The patient was seropositive to HTLV-1 using Western Blot testing and was seronegative for the Human Immunodeficiency Virus (HIV). Hepatitis B and C and Cytomegalovirus viral capside antigen (CMV VCA) IgM antibodies were not detected. EBV nuclear antigen IgG was positive, this pattern is characteristic of past EBV infection. An excisional biopsy from a left cervical lymph node showed a diffuse, large-cell B-cell morphology. Bone marrow aspiration and biopsy revealed a normocellular marrow showing trilineage hematopoiesis, without evidence of lymphoma or other morphological abnormalities.

Automated immunohistochemistry studies were performed on paraffin-embedded tissue sections. The tumor cells were positive for CD20 (Dako, Carpinteria, CA; dilution 1:100; Figure 1), PAX5 (Santa Cruz Biotechnology, Santa Cruz, CA; dilution 1:100) and MUM1 (Santa Cruz Biotechnology; dilution 1:200; Figure 2) and negative for CD10 (Novocastra; Newcastle upon Tyne, UK; dilution 1:10), BCL-6 (Dako; dilution 1:10), CD30 (Novocastra; dilution 1:100) and LMP-1 (Dako; dilution 1:100). Automated chromogenic in situ hybridization (CISH) for EBER was performed according to the manufacturer's protocol (Dako), and showed positive nuclear expression in tumoral cells (Figure 3).

**Figure 1:**
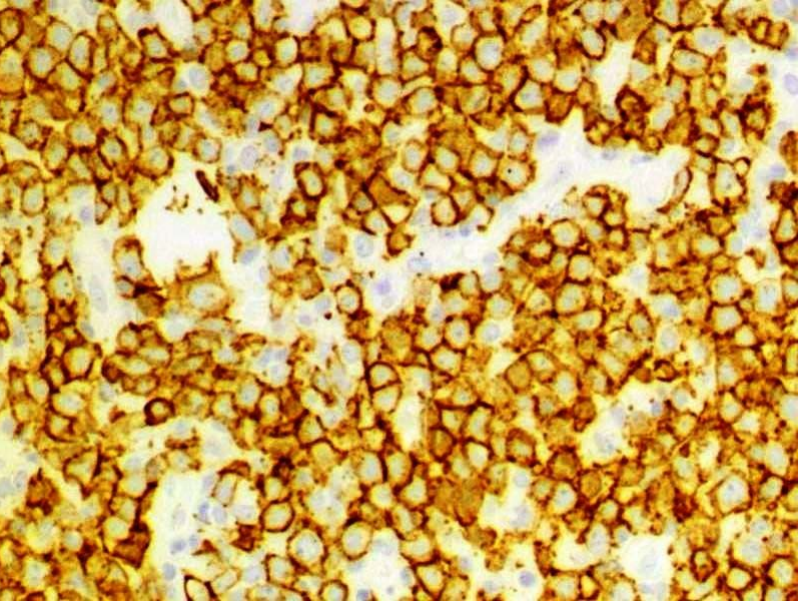
**Immunohistochemical expression of CD20**. CD20 is a pan-B-cell marker, demonstrating the B-cell lineage of this lymphoma (100×)

**Figure 2:**
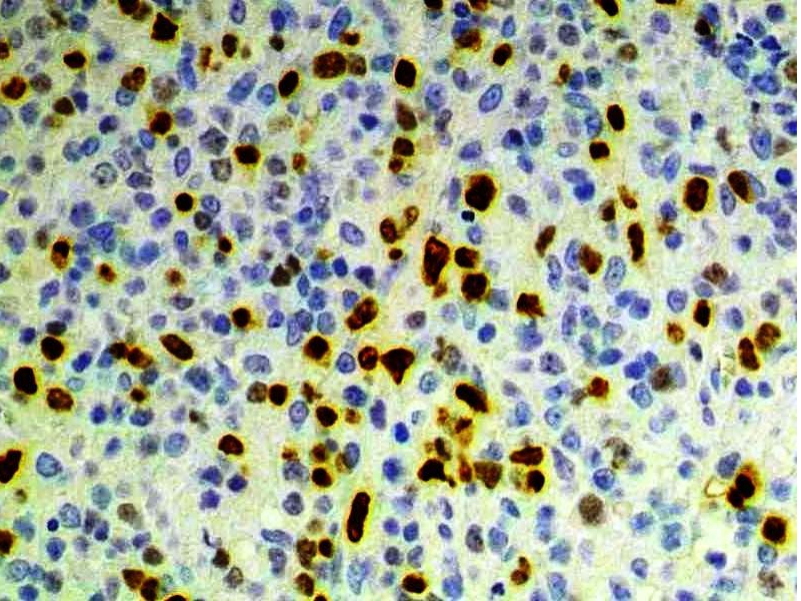
**Immunohistochemical expression of MUM1**. MUM1 is a plasma cell marker and, in DLBCL, is consistent with a non-germinal center subtype. DLBCL with a non-germinal center profile have been associated with worse survival (100×)

**Figure 3:**
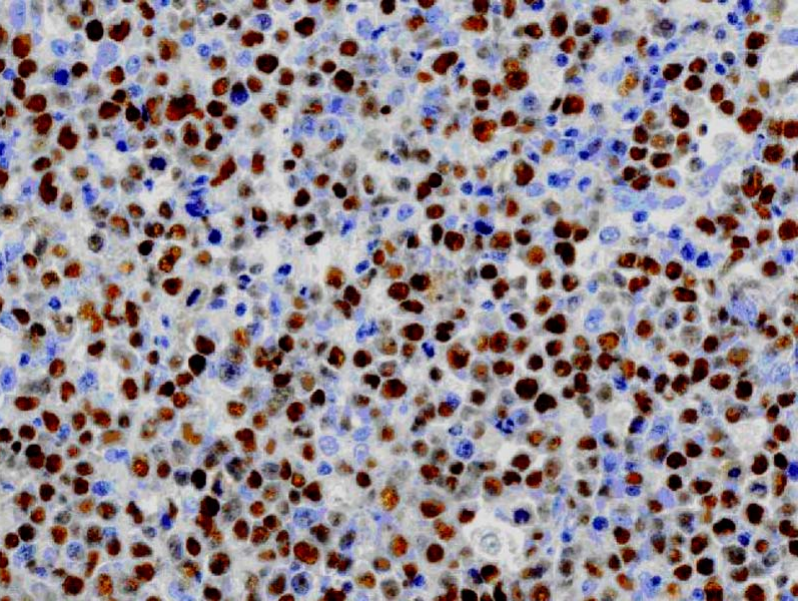
**Immunohistochemical detection of EBV-encoded RNA (EBER)**. Nuclear expression is demonstrated through automated chromogenic in situ hybridization (75×)

Before treatment, written consent was obtained from the patient, who was then defined as stage IIA DLBCL with a low-risk International Prognostic Index (IPI) score of 1 out of 5 (i.e. age older than 60 years). Treatment was started with cyclophosphamide, doxorubicin, vincristine and prednisone (CHOP) every 21 days with 25% dose-reduction of cyclophosphamide and doxorubicin with granulocyte-colony stimulating factor (G-CSF) support given patient's age. Four cycles of dose-reduced CHOP were administered. The patient achieved a complete response by radiologic criteria after the fourth cycle of treatment. Before the fifth cycle, the patient developed an interstitial pneumonia with increased serum LDH levels. *Pneumocystis jirovecci *pneumonia (PJP) was confirmed by immunofluorescent staining. Serology for *Legionella*, *Chlamydia *and *Mycoplasma *were negative. The patient died in the Intensive Care Unit 25 days after onset of PJP, 5 months after his lymphoma diagnosis.

EBV is a herpesvirus with demonstrated B-cell lymphotropism. EBV infection starts by attachment of the virus to the CD21 antigen; this initial step prepares the B-lymphocyte for EBV infection and is characterized by increased production of IL-6 and mRNA along with blastic transformation and mobilization of calcium. EBV is then inserted into the nucleus where it, episomally, acquires a circle-shaped configuration. EBV nuclear antigens (EBNA-LP, -1, -2 and -3) are the first to be produced after infection; these products are essential for immortalization of the cell and upregulation of the expression of other molecules and genes such as the latent membrane proteins (LMP-1 and -2). EBNAs also upregulate c-myc, which is a well-known human oncogene associated with cell proliferation. LMPs increase expression of BCL-2 and drive the cell into a latent state, which is maintained by the production of EBV-encoded RNA (EBER-1 and -2). In this way, EBV-infected B-cells enter the resting phase avoiding immunosurveillance but, due to their activated phenotype, more prone to develop secondary oncogenic changes [11]. In the present case, the serologic studies are consistent with a prior EBV infection and the immunohistochemical studies showed expression of EBER. The presence of EBNA-2 was observed in 28% of the cases of age-related EBV-associated lymphoproliferative disorder reported by Oyama and colleagues [12], which is indicative of a type III EBV latency, similar to the one observed in some cases of HIV-associated [13] and post-transplant lymphoproliferative disorders [14]. Genetic factors could also play a role in the development of EBV-associated lymphoma; it has been suggested that a genetically determined susceptibility, possibly based on certain HLA types, results in an abnormal response to primary EBV infection in certain parts of Asia [15]. These variations in HLA phenotype may provide a basis for the higher frequency of EBV-positive tumors among Asians. In addition, a recent study from Japan has shown that patients with EBV-associated NK/T-cell lymphomas, nasal type, have a low frequency of the HLA-A*0201 allele, suggesting the importance of this allele in cytotoxic T-lymphocyte responses [16].

HTLV-1 is a deltaretrovirus that infects a wide variety of cells, such as lymphocytes, monocytes and fibroblasts by virtue of its receptor, a commonly expressed transporter of glucose [17]. HTLV-1 induces a higher rate of production of infected cells rather than replicating itself, unlike HIV. A first step is to produce viral-associated proteins such as Tax, which is encoded in the pX region of the viral genome. Tax increases proliferation of virus-infected cells by accelerating all the phases of the cell cycle and renders the affected cells tolerant to a series of genetic and epigenetic changes [18]. The expression of Tax wears out as cells acquire the ability to proliferate independently. Due to its prolonged latency period of 40 to 60 years, HTLV-1 infected cells are more susceptible to acquire malignant phenotypes in a multistep process. Previous studies have indicated that the frequency of primary malignant neoplasms in HTLV-1 carriers is higher than in HTLV-1 seronegative non-Hodgkin lymphoma cases [19]. HTLV-1 infection is strongly associated to the development of ATLL and several Peruvian studies on clinical characteristics and outcomes of patients with ATLL have been reported [20]. Although HTLV-1 has not been associated with the development of B-cell lymphomas, HTLV-1 carriers with B-cell lymphoma tend to have a worse prognosis [21]. Theoretically, chronic HTLV-1 infection can cause T-cell dysfunction and B-cell proliferation inducing a particular state of immunosuppression favoring lymphomagenesis.

As people ages, their immune systems do not respond adequately to external pathogens or new antigens, such as immunizations or cancer. T-lymphocytes are greatly affected by this immune dysregulation state called immunosenescence since the number of naïve T-cells decrease in peripheral blood and lymph nodes [22], the distribution of T-cell population is altered [23] and the T-cell receptor repertoire becomes more limited [24]. The presence of persistent infections, such as EBV or HTLV-1 or other persistent antigens, such as cancer will also induce a phenomenon called immune exhaustion [25]. Other immunological changes associated with age include decrease in apoptosis [26] and elevation of levels of proinflammatory molecules [27]. This could potentially explain the increased incidence of cancers in the elderly. Furthermore, the detection of a type III EBV latency pattern speaks of a severe immunosuppression that could be partially explained by chronic HTLV-1 infection and/or immunosencescence. This theoretical relationship is currently unclear but plausible. To date, there are very few cases reporting the emergence of lymphoma in patients with coinfection by EBV and HTLV-1 [28-30].

Likely, HTLV-1 infection will cause chronic immunosuppresion and activate B-cell proliferation, favoring the development of EBV infection, which in turn will prepare the host for the development of B-cell lymphoma portending a worse prognosis. It seems intuitive that a patient with a combination of severe immunological impairment due to EBV and HTLV-1 infections, immunosenescence and chemotherapy would develop life-threatening opportunistic infections, although the occurrence of PJP in HTLV-1 carriers has seldom been reported in the medical literature [31,32]. Further research is needed to better understand the interaction between EBV and HTLV-1 in lymphomagenesis.

## Competing interests

The authors declare that they have no competing interests.

## Authors' contributions

BB, RS, FH, EC and LR provided clinical care to patient. PQ and DM carried out pathological studies. BB and JC prepared the manuscript. All the authors read and approved the final manuscript.
